# Collagenofibrotic Glomerulopathy: Three Case Reports in Brazil

**DOI:** 10.1186/1746-1596-4-33

**Published:** 2009-09-25

**Authors:** Renata DR Ferreira, Fabiano B Custódio, Camila SO Guimarães, Rosana RM Corrêa, Marlene A Reis

**Affiliations:** 1 Discipline of General Pathology, Biological Sciences Department, Triângulo Mineiro Federal University, Uberaba, Minas Gerais State, Brazil

## Abstract

**Background:**

We are reporting the first Collagenofibrotic Glomerulopathy (CG) in South America. So, this collagen type III glomerulopathy is not limited to Japan but may be found throughout the world.

**Case Reports:**

We describe three patients that presented some factors in common, such as sex, age and the presence of non-nephrotic proteinuria associated with microscopic hematuria. The findings with the immunofluorescence microscopy, of immunoglobulins, and components of the complement were usually negative. The picrosyrius staining showed the presence of reddish material in the mesangium, when it was seen under standard microscopy; however, when it was seen with birefringence, it became greenish under polarized light, showed the collagen found in this area of the glomerulus. The identification of CG was made through electronic microscopic scanning, and curved and disorganized fibers were found.

**Conclusion:**

These cases are the first from South America to be reported, and they are about an idiopathic renal disease that is not related to any specific races or locations. The reports contribute to a better understanding of this disease, which although not so prevalent, should be considered as an importantly differential diagnostic of cases of proteinuria.

## Background

Collagenofibrotic Glomerulopathy (CG) is a rare and recently defined entity characterized by deposition in the mesangial glomerulus and in the subendothelial space of type III collagen fibers [1]. It clinically manifests itself with proteinuria, hematuria, hypertension and variable degrees of renal failure in adults and children [2,3].

Type III collagen inside the basal membrane of the glomeruli is already part of the identification of another disease, known as Nail-Patella Syndrome. This syndrome is characterized by bone and nail abnormalities, associated with proteinuria of variable degrees. Publication of articles related to this new entity began in the late 70's, and it was made by a team of Japanese doctors who considered this disease to be either a variation of Nail-Patella Syndrome or a completely new one [4].

Based on the archive of renal biopsies at Nephopathology Service at General Pathology at the Federal University of Triângulo Mineiro (UFTM), we have identified three cases of CG that occurred from 2000 to 2007. There hadn't been any cases reported in South America until that time, since the great majority of cases had occurred in Japan [5].

## Case Presetation

### Case 1

**Figure 1:**
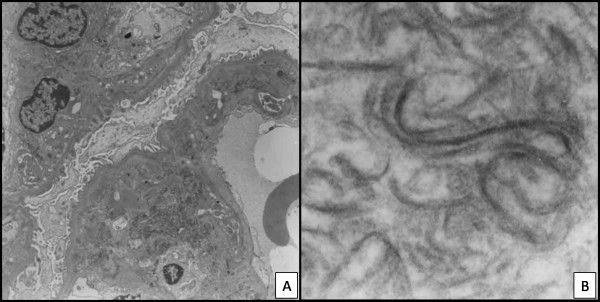
**Case 1 (2001)**: Electronic micrographs sections show expansion of the glomerular mesangium and subendothelial space by dense and curvilinear structures (banded fibrillar material. (Original magnification: {A}× 3000; {B} × 8500).

Female, 55 years old, hypertensive for the last 20 years. In the last 5 years, she had been showing microscopic hematuria associated with leukocyturia and cylindruria. The patient presented proteinuria (1.18 g/24 hours). Clearance of creatinine: 52 ml/min/1.73 m^2^. No changes to the clinical test. The patient underwent renal biopsy on December 12, 2000. One fragment was taken, because the patient showed severe hypertension during the performing of the biopsy. This fragment was processed by the electronic microscopy scanning, and consequently, there were no fragments for immunofluorescence microscopy. Renal Biopsy (semi-thin slices): There were eight glomeruli, and two of them were globally sclerotic. The other six glomeruli showed global expansion of the mesangium, thickening of capillary walls and no substantial hypercellularity. The capillary lumina were narrowed but not occluded. Foci of interstitial fibrosis and arteriolar hyaline deposits were found. Electronic microscopy scanning demonstrated expansion of the glomerular mesangium and subendothelial space by dense and curvilinear structures (banded fibrillar material). There were rare "calcium-like" deposits in subendothelial spaces. The dense lamina of the glomerular capillary basement membranes seemed normal (Figure 1A and 1B).

### Case 2

**Figure 2:**
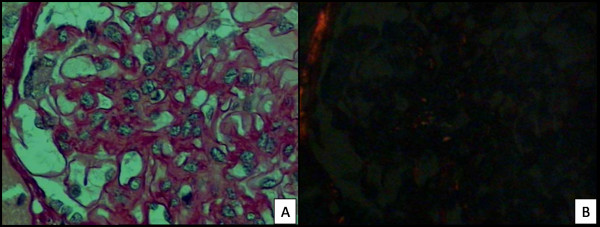
**Case 2 (2005)**: Section of a biopsy specimen stained with Picrosyrius shows mesangial expansion with reddish positive material (A) and under polarized light shows positive material with greenish birefringence (B). (High power).

Female, 21 years old, white, previously healthy and presenting no symptoms, no family background related to renal diseases. The patient presented proteinuria (1.6 g/24 hours) for a year, associated with microscopic hematuria. There is no information concerning renal functioning. The patient underwent a renal biopsy on May 31, 2005. Renal Biopsy: In the light microscopy, there were ten glomeruli, one of them was totally sclerotic. The rest presented mesangial hypercellularity which could go from mild to moderate, with apparent increase of the mesangial matrix. Staining with picrosyrius showed mesangial expansion with reddish positive material and with greenish birefringence under a polarized light microscopy. Tubules and interstice showed no changes (Figure 2A and 2B). Immunofluorescence microscopy: there were twenty glomeruli; negative to antibodies (also known as immunoglobulin, IgA, IgG and IgM) and to components of the complement (Ciq and C3). Electronic Microscopy Scanning: there were fourteen glomeruli, two of them were evaluated. There was hypercellularity in some mesangial axis, some amorphous or fibrillar deposits, some with irregular or curved shape. There was a change in the cytoplasm of the podocyte, with compressing of the cytoskeleton and foot process effacement. In some capillary walls, a thickening of the basal membrane was found, especially due to the enlargement of the subendothelial space.

### Case 3

**Figure 3:**
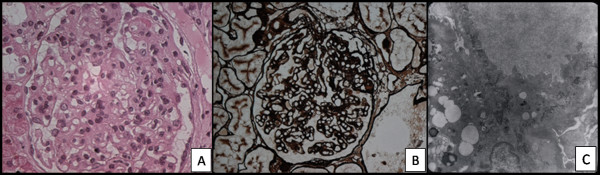
**Case 3 (2005) - Section of a biopsy specimen show endocapillary hypercellularity (A), especially by proliferation of mesangial cells**. In some capillary rings there are duplication of the basal membrane (B) (Silver, High power). Electronic micrographs of tannic acid-stained sections show increase of the matrix and fibrillar deposits (C) (Original magnification: × 4400).

female, 15 years old, with hypertension and initial edema of the limbs. Urine presented hematuria, piuria and proteinuria. The proteinuria (2.49 mg/24 hours) was associated with dyslipidemia (Total cholesterol: 426 mg/dl). Creatinine: 123,76 μmol/L. Urea: 31,77 mmol/L. High C3 and C4. Serology was negative for Hepatitis B and C. The patient was treated with inhibitor of angiotensin-converting enzyme, antagonist of the AT1 receptor for angiotensin II, statins, and corticosteroid, with partial response regarding the control of proteinuria. The patient underwent a renal biopsy on June 21, 2005. Renal Biopsy: In the light microscopy, there were ten glomeruli, all of them with endocapillary hypercellularity, especially by proliferation of mesangial cells. In four of the glomeruli, there were an increase of the mesangial matrix with in the segmental standard. In some capillary walls there were a duplication of the basal membrane. Immunofluorescence Microscopy: there were three glomeruli. IgM, Lambda and Kappa were positive and from mild to moderate levels in the capillary walls, global and diffuse. C3 was positive in the vascular walls. IgG, IgA and Ciq were negative. Electronic Microscopy Scanning: There were four glomeruli. Mesangial hypercellularity was found, followed by an often increase of the matrix and fibrillar deposits. The same deposits were in the subendothelial space, which resulted in the thickening of the capillary walls. The fibrillas presented irregular shape, and in some cases, curved shape, which may be compatible with type III collagen. There was a change in the cytoplasm of the podocytes, compressing of the cytoskeleton, and global foot process effacement (Figure 3A, B and 3C).

## Discussion

Clinic and morphologic findings through light and electronic microscopy scanning are related to that which is described as CG or Type III Collagen Glomerulopathy. CG has occurred both occasionally and within families. The cases with family background have shown the possibility of treating an autossomic recessive inherited disease [6,7]. The etiopathogenesis of this disease has been mentioned since the beginning of the cited publications, and it has also questioned CG as being a systemic disease or even only a variation that might come from glomeruli cells [8]. There is neither family background of the disease nor evidence of systemic diseases in any of the three reported cases. They presented some factors in common, such as sex, age (teenagers and young adults) and the presence of non-nephrotic proteinuria associated with microscopic hematuria.

The CG was initially considered a clinical variant of Nail-Patella Syndrome, but morphologic changes are different, and clinical findings and family studies have excluded this hypothesis. In contrast to Nail-Patella Syndrome glomerulopathy, diffuse glomerular changes are universally observed by light microscopy. They consist of marked enlargement of the glomerular tuft resulting from both expansion of the mesangial matrix and thickening of the cappillary walls [4,7], as observed in the three cases presented. The lesion may mimic diffuse thrombotic microangiopathy, or it may resemble type I membranoproliferative glomerulonephritis if mesangial hypercellularity is associated [6]. However, the hallmark of the disease is the presence of type III fibrillar collagen in the glomerular extracellular matrix observed by Electronic microscopy, and other differential diagnosis still may be established by immunofluorescence.

The findings under immunofluorescence microscopy to immunoglobulins and components of the complement are usually negative, when it comes to CG, as showed in the second case. Literature describes that conventional immunofluorescence is negative or shows focal non-specific deposits of immunoglobulins, mainly IgM, and complement components [4,6]. In the third case, IgM was positive in capillary walls; however, sparse positivity of IgM, IgG, C3 and other immunoreagents had already been reported which probably represents a flood of plasmatic proteins [9].

Picrosyrius staining was initially described in 1964 and it has been used to detect collagen in many tissues, including kidneys, and it is specific for type I and type III collagen. The red of Syria (component of the Picrosyrius) is a long coloring molecule which penetrates the folds of the collagen molecules and becomes birefrigent when observed under polarized light microscopy. Under standard microscopy light, it changes the color of the collagen into red and under polarized light, it exhibits a yellow-orange birefringence [10,11]. The picrosyrius staining, which was presented in the second case, showed the presence of reddish material in the mesangium, when it was seen under standard microscopy; however, when it was seen with birefringence, it became greenish under polarized light, which shows the collagen found in this area of the glomerulus. Picrosyrius staining has been used to study interstitial fibrosis and it seems to be useful in identifying collagen in the glomerulus, and also in helping the diagnosis of other glomerulopathies.

Electronic microscopy scanning is essential in the definitive diagnosis. In the majority of cases, the ultra structural findings provide evidence of collagen III. The fibers are in the mesangium and in the subendothelial space, but not in the subepithelial compartment or inside the basement membrane [12], as opposed to Nail-Patella syndrome in which the type III collagen is only inside of the basement membrane. In all three reported cases, the identification of CG was made through electronic microscopic scanning, and curved and disorganized fibers were found, which also created irregular bunches when they were cut lengthwise and with the shape of snakes or commas when the same were transversely cut. These ultra structural features distinguish the fibers of the collagen III from the fibers of regular collagen, which usually look like straight lines when cut lengthwise, and the same fibers look like circles when transversely cut.

## Conclusion

These cases are the first from South America to be reported, and they are about an idiopathic renal disease that is not related to any specific races or locations. The reports contribute to a better understanding of this disease, which although not so prevalent, should be considered as an importantly differential diagnostic of cases of proteinuria.

## Consent

Written informed consent was obtained from the families of patients for publication of this case report and any accompanying images. A copy of the written consent is available for review by the Editor-in-Chief of this journal.

## Competing interests

The authors declare that they have no competing interests.

## Authors' contributions

RDRF and FBC participated in the design of the study. CSOG helped to draft the final version of the manuscript. MAR and RRMC conceived of the study, and participated in its design and coordination and helped to draft the manuscript. All authors read and approved the final manuscript.

## References

[B1] Yoshioka K, Takemura T, Tohda M, Akano N, Miyamoto H, Ooshima A, Maki S (1989). Glomerular localization of type III collagen in human kidney disease. Kid Int.

[B2] Gibson W, More AR (1998). Glomerular pathology. Recents Advances. J Pathol.

[B3] Proesmans W, Van Dyck M, Devriendt K (2009). Nail-patella syndrome, infantile nephrotic syndrome: complete remission with antiproteinuric treatment. Nephrol Dial Transplant.

[B4] Dombros N, Katz A (1982). Nail patella-like renal lesions in the absence of skeletal abnormalities. Am J Kidney Dis.

[B5] Alchi B, Nishi S, Narita I, Gejyo F (2007). Collagenofibrotic Glomerulopathy: Clinicopathologic Overview of a Rare Glomerular Disease. Am J Kidney Dis.

[B6] Gubler MC, Dommergues JP, Foulard M, Bensman A, Leroy JP, Broyer M, Habib R (1993). Collagen type III glomerulopathy: a new type of hereditary nephropathy. Pediatr Nephrol.

[B7] Tamura H, Matsuda A, Kidogushi M, Matsumura O, Mitarai T, Isoda K (1996). A family with two sisters with Collagenofibrotic Glomerullonephropathy. Am J Kidney Dis.

[B8] Yasuda T, Imai H, Nakamoto Y, Ohtani H, Komatsuda A, Wakui H, Miura AB (1999). Collagenofibrotic Glomerulopathy: A Systemic Disease. Am J Kidney Dis.

[B9] Imbasciati E, Gherardi G, Morozumi K, Gudat F, Epper R, Basler V, Mihatsch MJ (1991). Collagen Type III Glomerulopathy: a New idiopathic Glomerular Disease. Am J Nephrol.

[B10] Grimm PC, Nickerson P, Gough J, McKenna R, Jeffery J, Birk P, Rush DN (1999). Quantitation of allograft IF and chronic allograft nephropathy. Pediatr Transplant.

[B11] Grimm PC, Nickerson P, Gough J, McKenna R, Stern E, Jeffery J, Rush DN (2003). Computerized image analysis of sirius red-stained renal allograft biopsies as a surrogate marker to predict long-term allograft function. J Am Soc Nephrol.

[B12] Ikeda K, Yokoyama H, Tomosugi N, Kida H, Ooshima A, Kobayashi K (1990). Primary glomerular fibrosis: a new nephropathy caused by diffuse intra- glomerular increase in atypical type III collagen fibers. Clinical Nephrol.

